# Undergraduate Students’ Perceived Stress Levels in Summer Term 2020 – A Comparison to Preceding Academic Terms

**DOI:** 10.3389/fpsyg.2021.672783

**Published:** 2021-06-02

**Authors:** Simone Antje Goppert, Maximilian Pfost

**Affiliations:** Department of Educational Research, Institute of Education, University of Bamberg, Bamberg, Germany

**Keywords:** perceived stress, higher education, e-learning, distance teaching, COVID-19

## Abstract

The COVID-19 pandemic tremendously affected teaching and learning in both schools and higher education settings. In Germany, university students had to shift from in-person group learning in lectures and seminars to new forms of e-learning and distance teaching. Even before COVID-19, stress was a common experience among university students, and these changes have reinforced students’ stress levels. Based on a sample of *n* = 110 German university students, this study explores whether students’ perceived stress levels in summer term 2020 differed from their perceived stress levels in preceding academic terms. The results show that students experienced lower levels of stress and higher levels of joy in summer term 2020 compared to preceding academic terms. Despite limitations in the interpretation of these findings, possible explanations, such as changes in academic and non-academic workload or decreased demands in university exams, are discussed.

## Introduction

University students often report substantial levels of perceived stress, especially during particularly challenging periods, such as the transition from school to university, which requires them to adapt to different forms of learning or develop a new identity as a university student (e.g., Perry et al., 2001; Denovan and Macaskill, 2017). The COVID-19 pandemic represents another such challenge for university teachers and students, as new forms of e-learning had to be established in a short period of time. Due to the need to adapt to these new forms of learning, it is likely that students’ perceived stress has changed. Therefore, this study aims to explore university students’ perceived stress levels in summer term 2020, which was strongly affected by the COVID-19 pandemic, in comparison with students in preceding academic terms.

## Theoretical Framework

### Stress Experience and Higher Education

Stress arises from an interaction between a person and the environment (see, for example, the transactional stress model; Lazarus and Folkman, 1984). A situation is perceived as stressful if it “is appraised by the person as taxing or exceeding his or her resources and endangering his or her wellbeing” (Lazarus and Folkman, 1984, p. 19). This means that experiencing stress is at least partially individual and subjective (Lazarus and Folkman, 1984; Lazarus, 1999; Fliege et al., 2001). Therefore, some people experience stress in a given situation, while others do not because they perceive and evaluate the same situation differently. It can be concluded that all kinds of changes in people’s lives, including changes and transitions in the learning environment, can lead to an increased level of stress (e.g., Clinciu, 2013; Sohail, 2013; Denovan and Macaskill, 2017). This assumption is also transferable to the summer term 2020 under COVID-19 and its change from classroom teaching to distance teaching and online learning.

Overall, stress is a common experience for university students: In Germany, 53.1% of students report a high level of stress during their studies and 41.6% report a medium level. Only 5.3% report a low stress level (Herbst et al., 2016; see Hudd et al., 2000, for comparable data on US college students). Therefore, students in Germany report higher levels of stress than working adults (Herbst et al., 2016). This may be explained by the high number of stressors students faces at university, such as a high workload due to a large amount of learning material, frequent exams, and/or worries about their future (Zeidner, 1992; Bedewy and Gabriel, 2015; Herbst et al., 2016). Furthermore, difficulties in time management can also lead to stress for students (e.g., balancing time for learning with time for other activities, including paid work; Herbst et al., 2016). Finally, stress can have detrimental consequences for university students, such as poor academic performance (Sohail, 2013), mental or psychosomatic symptoms, such as dissatisfaction, restlessness, search for distraction, sleeplessness, difficulty concentrating, or listlessness (Herbst et al., 2016). Thus, if even a normal term can cause a lot of stress for university students, what happens in a pandemic situation like COVID-19?

### Distance Learning During the COVID-19 Pandemic – A Stressful Challenge?

The COVID-19 pandemic led to enormous changes in learning conditions for school and university students worldwide within a short amount of time (e.g., Mishra et al., 2020). Universities had to digitalize their courses in order to continue to offer them to their students (Sahu, 2020). Studies on distance teaching and e-learning conducted ahead of COVID-19 show that distance teaching is a unique type of teaching and learning that is associated with different challenges than classroom teaching (Furlonger and Gencic, 2014), such as less face-to-face interaction with instructors and peers, technical difficulties, and less knowledge about course objectives (Furlonger and Gencic, 2014). In addition, 71% of students who regularly used an e-learning format reported dissatisfaction with the lack of connections to their fellow students (Song et al., 2004).

However, COVID-19 not only changed students’ learning but also changed their private life as a result of governmental action to handle the pandemic (e.g., contact restrictions and travel restrictions). Some students may also have lost their part-time jobs, whereas other students may have moved house in order to study from their parents’ home. Taken together, the shift from classroom teaching to distance teaching coupled with the challenges students faced in their private lives constitutes a process of change for students, which might therefore have had an impact on the students’ stress experience.

## The Present Research

The present study aims to explore differences in university students’ perceived stress in summer term 2020 in comparison with preceding academic terms. The study was conducted with undergraduate students from the University of Bamberg, Germany. The University of Bamberg is a public university with four faculties and more than twelve thousand students enrolled in academic year 2019/2020 (Ruppert, 2020). Prior to COVID-19, most courses in the field of education sciences were held in-person. However, in summer term 2020, during the COVID-19 pandemic, most courses switched to online/distance teaching. In addition to these changes in university learning, students faced many challenges in their daily lives in summer term 2020 due to the pandemic-related regulations and restrictions. Consequently, these may have led to changes in their psychological wellbeing (e.g., Zacher and Rudolph, 2021) and stress. Furthermore, university workload may have changed and had an impact on stress, but due to a lack of prior assumptions, all studies were exploratory.

## Materials and Methods

### Procedure

Data were collected as part of a broader evaluation of university students’ learning at the University of Bamberg, Germany. This evaluation project aims to analyze the learning conditions and learning outcomes of university students within education sciences, with a particular emphasis on self-regulated learning. In this paper, we focus on education students enrolled in an introductory psychology lecture. This course was evaluated every semester since the winter term 2018/19. In the second half of the semester, students were invited to respond to a questionnaire that included a question on their perceived stress level. The introductory lecture has two parts, one offered in the winter and one in the summer term. Students are free to decide whether to begin the lecture in the winter or summer term. Consequently, some students participated in the study twice. In order to avoid dependencies within the data and consider every student just once, we conducted a random selection procedure. Therefore, just one of the two available questionnaires for each student with multiple participation time points were considered in our analyses. In summer term 2020, data collection started on June 23 and ended on July 9 (17 days).

### Sample

Eighty-four university students participated once and 26 university students participated twice in our study. This resulted in a total sample of 110 university students, as students who participated twice were only considered once in our analyses (see procedure). On average, the participating students were 21.64 years old and in their 1.87 semester of studies. Eighty-three percent of the students were female. Within the analyzed sample, 31 (28%) students responded to our questionnaire in winter term 2018/19, 33 (30%) students in summer term 2019, 26 (24%) students in winter term 2019/2020, and 20 (18%) students in summer term 2020. As the study focused on differences between students participating in the study in summer term 2020 compared to preceding terms, we checked for differences in age (preceding terms/summer term 2020: *M* = 21.45/22.45), semester (*M* = 1.83/2.05), and proportion of female students (82%/85%) between these two groups. None of the differences were significant.

### Measures

#### Perceived Stress

Students’ subjectively experienced stress was assessed with 20 items from the German short version of the *Perceived Stress Questionnaire* (PSQ, Fliege et al., 2001). Using four scales with five items per scale, the PSQ focuses on current subjectively perceived stress on a cognitive and affective level. In the scale introduction, students were asked to indicate how often these statements apply to their lives in general during the (online) lecture period. Each item consisted of a statement that had to be rated on a four-point Likert scale (1 = hardly ever, 2 = sometimes, 3 = frequently, and 4 = most of the time). Current sorrows and fear about the future (for example, “I have fears about the future”) are summarized in the *worries* scale (Cronbach’s *α* = 0.86). The *tension* scale encompasses difficulty relaxing or feelings of exhaustion (e.g., “I have difficulty relaxing”; Cronbach’s *α* = 0.82). Perceived external *demands*, such as time pressure or having too much to do, are summarized in the demands scale (e.g., “I have too many things to do”; Cronbach’s *α* = 0.77). Contrary to the first three scales, the *joy* scale focuses on positive experiences, such as having fun or feelings of security and protection (e.g., “I have the feeling that I am doing things I really like to do”; Cronbach’s *α* = 0.80). As the items for this scale were formulated in a positive way, low scale scores, representing the absence of joy, indicate higher levels of perceived stress. Scale scores were estimated by calculating the arithmetic mean. There were hardly any missing responses. However, in the case of missing responses to single items, the arithmetic mean of the remaining items was taken.

#### Time Spent Attending University Courses

In order to estimate the time students typically spent attending university courses, we asked the following question: “How many hours have you spent attending university courses on average in a typical week this semester?” Responses were provided in an open-response format in hours per week. In summer term 2020, the question was changed slightly, as we asked about “online university courses” rather than “university courses.”

#### Additional Questions in Summer Term 2020

Some additional questions concerning specifics of the online learning situation were added to the questionnaire used in summer term 2020. With respect to *changes in stress level*, we asked “In comparison to a semester with in-person teaching, I have the impression that my stress level has … due to online teaching.” Responses were provided on a seven-point Likert scale (1 = strongly decreased, 4 = remained equal, and 7 = strongly increased). Using the same response options, we also asked about *changes in individual workload* (“In comparison to a semester with in-person teaching, I have the impression that my workload has … due to online teaching.”).

### Data Analytic Strategy

First, descriptive statistics and correlations were estimated using IBM SPSS Version 26. In order to analyze differences in perceived stress, a dummy variable distinguishing between semesters with in-person teaching (winter term 2018/19, summer term 2019, and winter term 2019/20; coded as 0) and the semester of online teaching (summer term 2020; coded as 1) was generated. In addition, effect sizes *d* were estimated based on the standard deviation of all analyzed students, and differences were tested using Welch’s *t*-test.

In order to test for differences in perceived stress, a latent variable based on the manifest stress scales was estimated using Mplus 8.4 (Muthén and Muthén, 1998–2017). The dummy variable for in-person vs. online teaching was used as a predictor variable. In a second model, we tested whether the effect of in-person vs. online teaching was mediated by time spent attending university courses. In the latent models, we used an MLR estimator and treated missing data with FIML. Model fit was evaluated based on recommendations by Schermelleh-Engel et al. (2003).

## Results

### Descriptive Statistics

Means and standard deviation of the PSQ scales and the time students spent attending university courses are presented in Table 1. Correlations are shown in Table 2. In comparison with the preceding terms, the descriptive statistics show the lowest perceived stress scores on the worries, tension, and demands scales and the highest scores on the joy scale in summer term 2020, the period of online teaching. These descriptive differences were significant for the worries and joy scales. Furthermore, students spent a lower number of hours attending university courses in summer term 2020 compared to the preceding terms. Strong correlations among the worries, tension, and demands stress scales were found, whereas joy was negatively correlated with these scales. The time students spent attending university courses was not significantly related to the PSQ scales.

**Table 1:** Means and standard deviations.

	All terms (*n* = 110)	Winter term 2018/19 (*n* = 31)	Summer term 2019 (*n* = 33)	Winter term 2019/20 (*n* = 26)	Summer term 2020 (*n* = 20)	Diff. all terms	Contrast	
	*M* (*SD*)^1^	*M* (*SD*)	*M* (*SD*)	*M* (*SD*)	*M* (*SD*)^1^	Value of *p*	*d*	Value of *p*
PSQ-worries	2.15 (0.71)	2.14 (0.55)	2.36 (0.78)	2.18 (0.78)	1.80 (0.61)	*p* < 0.05	−0.61	*p* < 0.01
PSQ-tension	2.22 (0.60)	2.23 (0.62)	2.41 (0.61)	2.12 (0.58)	2.00 (0.53)	*ns*	−0.43	*ns*
PSQ-demands	2.21 (0.59)	2.29 (0.60)	2.33 (0.60)	2.09 (0.61)	2.06 (0.54)	*ns*	−0.32	*ns*
PSQ-joy	2.69 (0.58)	2.64 (0.58)	2.60 (0.63)	2.64 (0.54)	2.96 (0.48)	*ns*	0.59	*p* < 0.01
Courses (h)	14.39 (5.64)	15.23 (4.67)	12.97 (4.18)	16.92 (5.77)	12.05 (7.58)	*p* < 0.05	−0.50	*ns*

1 Transformed PSQ scale scores ((x − 1)/3) to compare with Sieber et al. (2020) are for all terms (row 1): worries: *M* = 0.38; tension: *M* = 0.41; demands: *M* = 0.40; and joy: *M* = 0.56 and for summer term 2020 (row 5): worries: *M* = 0.27; tension: *M* = 0.33; demands: *M* = 0.35; and joy: *M* = 0.65. Diff. all terms indicates values of *p* on robust ANOVA (Welch’s *t*-test) between all terms (four groups). Contrast indicates effect sizes of the difference between summer term 2020 and the mean of the three remaining terms (two groups); estimation of effect size *d* is based on the standard deviation of all students across all terms; and furthermore, within the last column, values of *p* on Welch’s *t*-test for this contrast are provided.

**Table 2:** Correlations.

S. No.		1	2	3	4	5
1.	PSQ-worries	–				
2.	PSQ-tension	0.65^**^	–			
3.	PSQ-demands	0.54^**^	0.68^**^	–		
4.	PSQ-joy	−0.50^**^	−0.47^**^	−0.27^**^	–	
5.	Courses (h)	−0.11	−0.13	−0.09	−0.05	–

** *p* < 0.01.

### Latent Variable Analyses

In order to test for differences in perceived stress while taking measurement error into account, a latent variable approach was followed. First, we estimated a latent stress variable based on manifest scale scores for all four PSQ scales. The joy scale was inverted so that all scales pointed in the same direction. With the exception of the first variable, factor loadings and means were not constrained (τ-congeneric model; Steyer, 2001; Steyer and Eid, 2001). The implied variance-covariance structure did not fit the empirical variance-covariance structure well, resulting in a low model fit (*χ*^2^ = 10.90, *df* = 2, *p* < 0.05; RMSEA = 0.20; CFI = 0.93). This low model fit was primarily due to the joy scale, which did not correspond well with the other three PSQ scales. Therefore, a latent stress variable based on the three remaining PSQ scales was estimated. In order to ensure model identification, all factor loadings were set to be equal (essential τ-equivalent model; Steyer, 2001; Steyer and Eid, 2001). Model fit was acceptable to good (*χ*^2^ = 3.06, *df* = 2, *ns*; RMSEA = 0.07; CFI = 0.99).

Next, a dummy variable indicating whether students participated at the study in summer term 2020 (online teaching; coded as 1) or before summer term 2020 (in-person teaching; coded as 0) was added (*χ*^2^ = 6.17, *df* = 4, *ns*; RMSEA = 0.07; CFI = 0.98). The dummy variable negatively predicted the latent perceived stress variable (*B* = −0.27, *SE* = 0.12, *p* < 0.05; standardized parameter *β* = −0.21). Therefore, students indicated lower perceived stress in summer term 2020 compared to the preceding terms.

**Figure 1:**
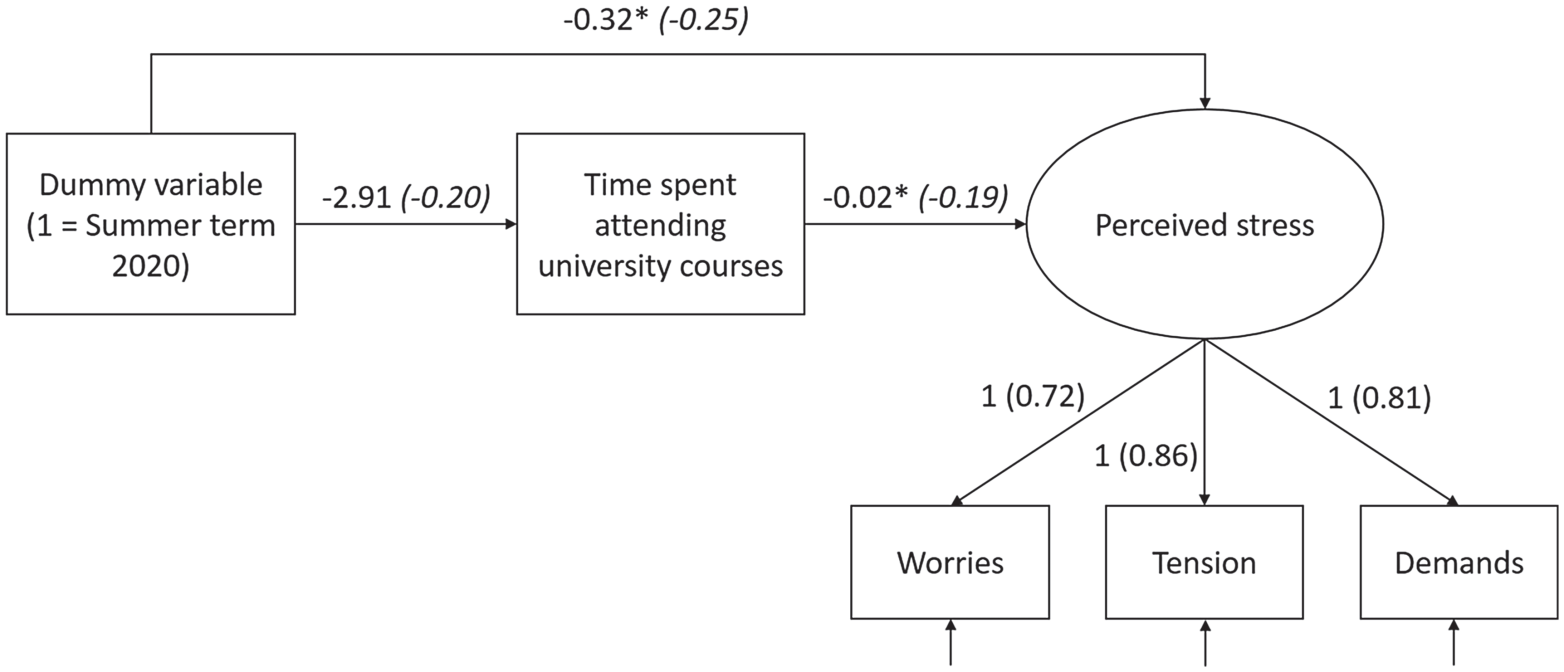
Latent variable model. In addition to the unstandardized coefficients, standardized coefficients are shown in brackets. ^*^p < 0.05.

Finally, we estimated a model that additionally took students’ time spent attending university courses as a possible mediator into account (Figure 1). Model fit was satisfactory (*χ*^2^ = 6.59, *df* = 6, *ns*; RMSEA = 0.03; CFI = 1.00). The results show that the dummy variable indicating participation in summer term 2020 still negatively predicted perceived stress. However, the dummy variable did not predict the time students spent attending university courses. The time students spent attending university courses was negatively related to students’ perceived stress. The more hours students spent attending university courses, the less stress these students perceived. Finally, the results do not reveal any indirect effect of the dummy variable indicating participation in summer term 2020 on perceived stress via time spent attending university courses (*B*_Ind_ = 0.05, *SE* = 0.04, *ns*; standardized parameter *β*_Ind_ = 0.03).

### Additional Analyses

In order to check the PSQ results, students in summer term 2020 provided a self-rating on perceived differences in their stress level and workload between online and in-person teaching. Concerning stress level, a mean of *M* = 3.85 (*SD* = 1.50; 95% CI from 3.24 to 4.50) was found. This is slightly below the scale’s theoretical mean. Therefore, despite substantial individual variability, students did not report increased stress due to online teaching on average. For workload, a mean of *M* = 4.90 (*SD* = 1.45; 95% CI from 4.25 to 5.50) was found, which slightly exceeded the scale’s theoretical mean. Consequently, students indicated having a higher workload on average.

## Discussion

### Findings and Practical Implications

Contrary to our assumptions, the results showed that students did not report increased stress in summer term 2020 compared to preceding academic terms. Rather, the results indicate that the students experienced fewer worries and more joy in their studies. Our conclusion is further supported by a comparison of the scale means with other studies using the PSQ (e.g., Sieber et al., 2020). Furthermore, our data showed no sign of increasing workload due to increasing hours spent in university courses, and consequently, time spent by students in university courses did not prove to be a significant mediator variable. With respect to these results concerning perceived stress and wellbeing, it is important to note that there were some positive changes to university learning conditions for students during and after the first wave of the COVID-19 pandemic in addition to the negative changes. For example, students no longer needed to get up early to travel to the university for class, and recorded lectures gave students more flexibility in managing their time. Furthermore, the maximum number of failed exam attempts allowed before expulsion from a study program was suspended (normally there are only two failed attempts), meaning that students faced less pressure to perform. Non-university-related factors could also have had a positive influence on students’ stress experience, such as working fewer hours in a part-time job or fewer opportunities for leisure activities, which gave students more time for university and led to less stress in the leisure and work domains. Finally, the time period of data collection coincided with a general trend of decreasing infection rates in Germany (Robert Koch Institute, 2020), which may have had a positive impact on students’ psychosocial wellbeing.

All in all, it seems that distance learning and teaching in summer term 2020 did not necessarily negatively influence students’ stress experience. However, our findings are merely a one-time snapshot of how COVID-19 changed students’ stress experience and psychological wellbeing; further monitoring of students’ stress and wellbeing in the forthcoming terms after summer term 2020 still seems worthwhile.

### Limitations and Future Directions

A first limitation of our study is the small sample size for summer term 2020. The small and uneven sample leads to inferential statistics with large standard errors and estimates of low precision. Furthermore, as our sample consisted solely of education sciences students, caution is warranted when generalizing the results to other fields of study. Indeed, access to the university environment (e.g., laboratories) is more important for other disciplines (e.g., chemistry and medicine). Finally, our study did not ask students about coping strategies. Thus, it is not possible to provide detailed reasons for our findings.

## Conclusion

Stress research is an important topic for universities. Based on the previous stress research, we initially assumed that the COVID-19 pandemic and resulting changes in university teaching would have a negative impact on students’ stress experience. This was not supported by our data. Instead, it must be assumed that students have the necessary coping strategies to deal with the consequences of changing learning conditions during the pandemic. Going beyond these findings, the changes in teaching and learning necessitated due to COVID-19 can be seen as an opportunity to develop new forms of teaching and learning at universities (e.g., lectures in audio and video podcasts), which can hopefully enrich university education in the long term.

## Data Availability Statement

The datasets presented in this article are not readily available because of privacy restrictions. Requests by qualified researchers to access the minimal set of variables used in the study should be directed to the authors. Requests to access the datasets should be directed to simone.goppert@uni-bamberg.de or maximilian.pfost@uni-bamberg.de.

## Ethics Statement

Ethical review and approval were not required for the study on human participants in accordance with the local legislation and institutional requirements. The participants provided their written informed consent to participate in this study.

## Author Contributions

All authors were involved throughout the study, wrote the manuscript, and approved the final manuscript. SG organized data collection. MP performed the statistical analyses presented in the final manuscript.

### Conflict of Interest

The authors declare that the research was conducted in the absence of any commercial or financial relationships that could be construed as a potential conflict of interest.
